# Supplementary material for: Activation of the ROS/CncC Signaling Pathway Regulates Cytochrome P450 CYP4BQ1 Responsible for (+)-α-Pinene Tolerance in Dendroctonus armandi
Source: Int J Mol Sci. 2022 Sep 30;23(19):11578. doi: 10.3390/ijms231911578 (PMC9569712; doi:10.3390/ijms231911578)
Supplement: Supplementary file 1 [file ijms-23-11578-s001.zip › ijms-1935561-supplementary.pdf]

Supplementary Material

Table S1 Primer sequences used in the research

	Sequence (5′ → 3′)		purpose
	Forward	Reverse	
CYP4BQ1	CGACGATGGCACTAAGAGGA	GAAACTAATGGCTGAAGCTG	qPCR
	taatacgactcactatagggGCAAGCAAGTAGACAAGGAT	taatacgactcactatagggCAGTAGCAAGTCAAGAAAGG	RNAi
CYP4BQ3	AGTGGGGGCGAAGAGTAAAA	CGCAAAACTAAGGGCTGAAG	qPCR
CYP4BG4	CTCTGGTCTACTCCCTGTCT	AGCATTTTCATAATTTTCCT	qPCR
CYP4BR4	CATCCGCTATTCTTCCGCTT	CCCTGTAGGTTCGCTTCTAA	qPCR
CncC	CACTCAACAATGACGACGGT	CCAAGGAGAAGCCCAGATCC	qPCR
	taatacgactcactatagggATTGGAGACGATTTCGATAT	taatacgactcactatagggGTTCACTGCTGTTTGTTAGG	RNAi
Keap1	AATACAGATACATGGACGGA	GATGAAGATGAACAAGGAGA	qPCR
Maf	TTGGTCAGCATCTCTGTTCG	GCCATTCTTGCGTCTTTTCG	qPCR
GFP	taatacgactcactatagggATGGTGTTCAATGCTTTTCA	taatacgactcactatagggCTCTCTTTTCGTTGGGGTCT	RNAi

Note: T7 promoter sequences in the dsRNA synthesis primers were indicated in lowercase letters.

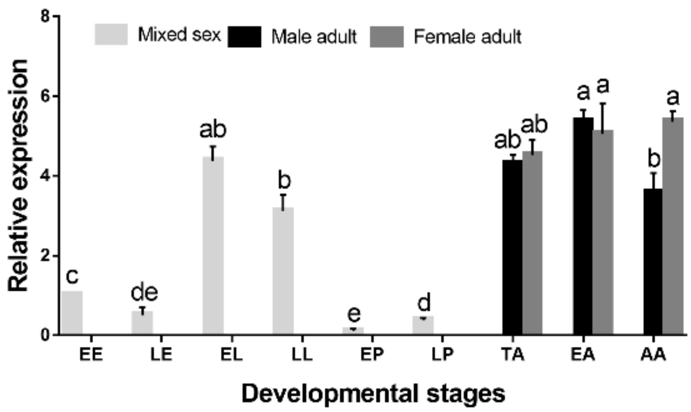

Figure S1. Relative mRNA expression levels of *CYP4BQ1* in different developmental stages of *D. armandi*. The relative expression levels were normalized with *β-actin* and

CYP4G55. Different lowercase letters indicate significant differences at $P < 0.05$. (ANOVA, Turkey test). All values are mean ± SE, $n = 3$. EE, early egg; LE, late egg; EL, early larvae; LL, late larvae; EP, early pupae; LP, late pupae; TA, teneral adult; EA, emerged adult; AA, attacking adult.

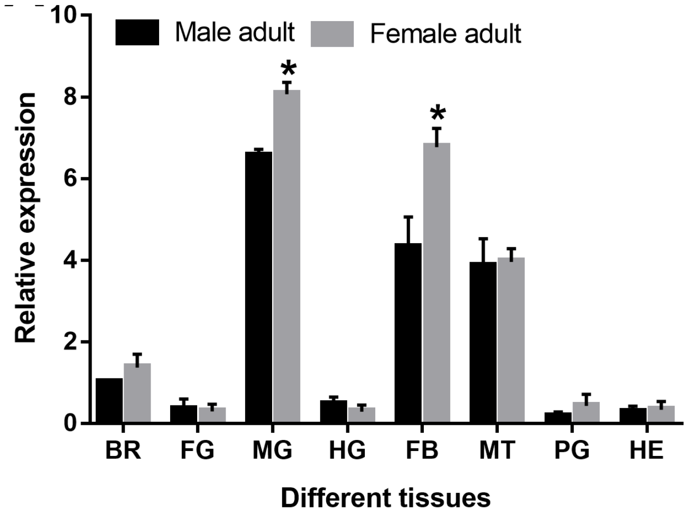

Figure S2. Relative expression levels of emerged adults of *CYP4BQ1* in different tissues of *D. armandi*. The relative expression levels were normalized with *β-actin* and *CYP4G55*. The asterisk indicates a significant difference between female and male expression levels (**P* <0.05, independent Student's Test). All values are mean ± SE, n = 3. BR, brain; FG, foregut; MG, midgut; HG, hindgut; FB, fat body; MT, Malpighian tubules; PG, pheromone gland; HE, hemolymph.

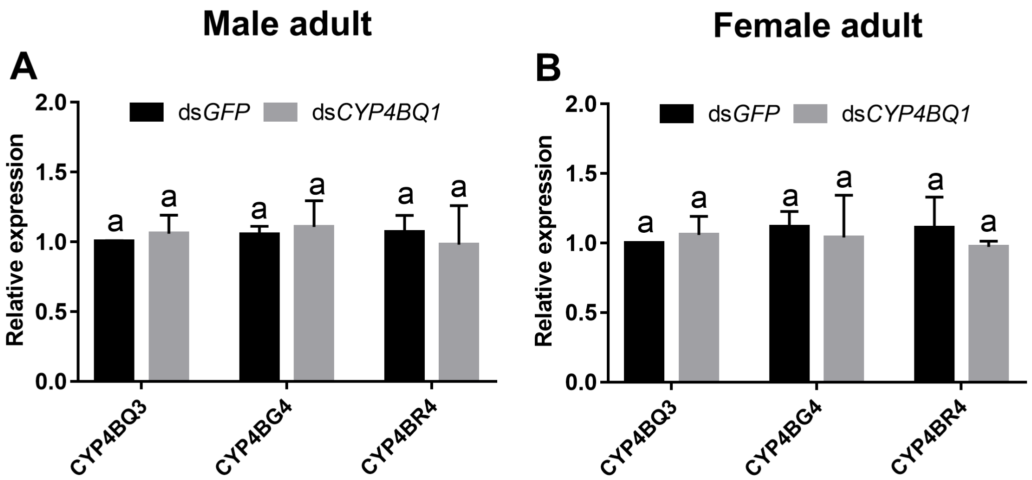

Figure S3. Relative expression levels of three CYP4B subfamily genes (*CYP4BQ3*, *CYP4BG4* and *CYP4BR4*) in *D. armandi* emerged male (**A**) and female (**B**) adults after injected dsRNA at 72h. The relative expression levels were normalized with *β-actin* and *CYP4G55*. Different letters indicate significant differences at $P < 0.05$. All values are mean ± SE, $n = 3$.
